# Development of a Broth Microdilution Method for Exebacase Susceptibility Testing

**DOI:** 10.1128/AAC.02587-20

**Published:** 2021-06-17

**Authors:** Jun T. Oh, Jane E. Ambler, Cara Cassino, Raymond Schuch

**Affiliations:** a ContraFect Corporation, Yonkers, New York, USA

**Keywords:** direct lytic agents, lysin, endolysin, exebacase, MIC, CLSI, CF-301

## Abstract

Exebacase (CF-301) belongs to a new class of protein-based antibacterial agents, known as lysins (peptidoglycan hydrolases). Exebacase, a novel lysin with antistaphylococcal activity, is in phase 3 of clinical development. To advance into the clinic, it was necessary to develop an accurate and reproducible method for exebacase MIC determination. The Clinical and Laboratory Standards Institute (CLSI) reference broth microdilution (BMD) method using cation-adjusted Mueller-Hinton broth (CAMHB) produced trailing MIC endpoints, and exebacase activity was diminished when frozen BMD panels were used. A modified BMD method was developed using CAMHB supplemented with 25% horse serum and 0.5 mM dl-dithiothreitol (CAMHB-HSD). Preliminary quality control (QC) ranges for Staphylococcus aureus ATCC 29213 of 0.25 to 1 μg/ml and for Enterococcus faecalis ATCC 29212 of 16 to 64 μg/ml were determined based on the results of a CLSI M23-defined MIC QC tier 1 study. These preliminary QC ranges validated the MIC data generated from a systematic study testing a discrete S. aureus strain collection using CAMHB-HSD to investigate the impact of parameters known to influence susceptibility test results and to evaluate the exebacase MIC distribution against clinical S. aureus isolates. Presentation of these data led to the CLSI Subcommittee on Antimicrobial Susceptibility Testing (AST) approval of the use of CAMHB-HSD to determine exebacase susceptibility and commencement of a multilaboratory (tier 2) QC study. Use of a standard BMD method and concomitant QC testing provides confidence in the assessment of test performance to generate accurate and reproducible susceptibility data during antibacterial drug development.

## INTRODUCTION

A promising new therapeutic approach to killing antibiotic-resistant bacterial pathogens is based on the use of direct lytic agents (DLAs), including lysins, which represent a new antimicrobial modality currently in clinical development (1). Lysins are recombinantly produced cell wall peptidoglycan hydrolytic enzymes that elicit rapid cleavage of the cell wall and resultant osmotic lysis (1, 2). Exebacase is an antistaphylococcal lysin with the following microbiological attributes: (i) rapid, targeted bactericidal activity, (ii) the ability to eradicate staphylococcal biofilms, (iii) synergy with antistaphylococcal antibiotics, (iv) a low propensity for the development of resistance, (v) no cross-resistance with antibiotics, and (v) an extended *in vitro* and *in vivo* postantibiotic effect (3–7).

Antibacterial susceptibility testing is the mainstay of antibacterial drug development, providing important data to assess the potential clinical efficacy of an investigational drug for the intended indication(s). While for many traditional antibiotics the CLSI reference broth microdilution (BMD) method is used to assess susceptibility, it is recognized that for some drugs, in particular those targeting difficult-to-treat multidrug-resistant (MDR) bacteria, modification of the reference method is necessary (8–10). Such modifications include supplementation of cation-adjusted Mueller-Hinton broth (CAMHB) with Ca^2+^ (for daptomycin [11, 12]) or polysorbate (Tween) 80 (for dalbavancin and oritavancin [12, 13]) and the use of iron-depleted CAMHB prepared with Chelex 100 resin (for cefiderocol [12, 14]). Tigecycline and omadacycline susceptibility testing requires the use of freshly prepared medium (≤12 h), noting that panels may be frozen for later use (14, 15). Given the recent rise in drugs that necessitate a modified BMD method, it is likely that drugs requiring medium modifications will become more commonplace, in particular with future investigational nontraditional antibacterial agents having novel clinical development programs to address unmet medical needs with regard to specific MDR pathogens.

A standardized MIC assay for a therapeutic lysin has not previously been described, nor has a protein-based direct lytic agent (e.g., a nonantibiotic) been evaluated by a recognized standards development organization, such as CLSI. Before conducting clinical trials in the United States, drug sponsors must describe the methods used for generating susceptibility data by referencing a recognized standards development organization, such as CLSI, or evaluate susceptibility by other methods, including modification of the method. Herein, exebacase MIC data generated using the CLSI reference BMD method are presented and compared with analogous data generated using CAMHB supplemented with 25% horse serum and 0.5 mM dl-dithiothreitol (CAMHB-HSD) against a discrete collection of Staphylococcus aureus isolates. Preliminary MIC quality control (QC) ranges for S. aureus ATCC 29213 and Enterococcus faecalis ATCC 29212 were then identified in accordance with CLSI M23-A4 using the proposed modified method. The preliminary QC ranges served to validate the exebacase MIC distribution generated by testing 149 S. aureus clinical isolates as well as the MIC results of an M23-defined systematic study conducted with CAMHB-HSD to investigate the impact of parameters known to influence susceptibility test results. Based on these studies, the CLSI Subcommittee on Antimicrobial Susceptibility Testing (AST) approved the use of CAMHB-HSD to determine exebacase MICs. A multilaboratory MIC (tier 2) QC study was subsequently initiated to establish expected QC ranges for the new BMD method (30). The BMD method for the determination of exebacase susceptibility and the expected QC ranges for exebacase, which based on the tier 2 study results were defined as 0.25 to 2 μg/ml for S. aureus ATCC 29213 and 8 to 64 μg/ml for E. faecalis ATCC 29212, have been published in CLSI document M100 (12).

## RESULTS

### Limitations of the CLSI reference BMD method for exebacase MIC determination.

**FIG 1:**
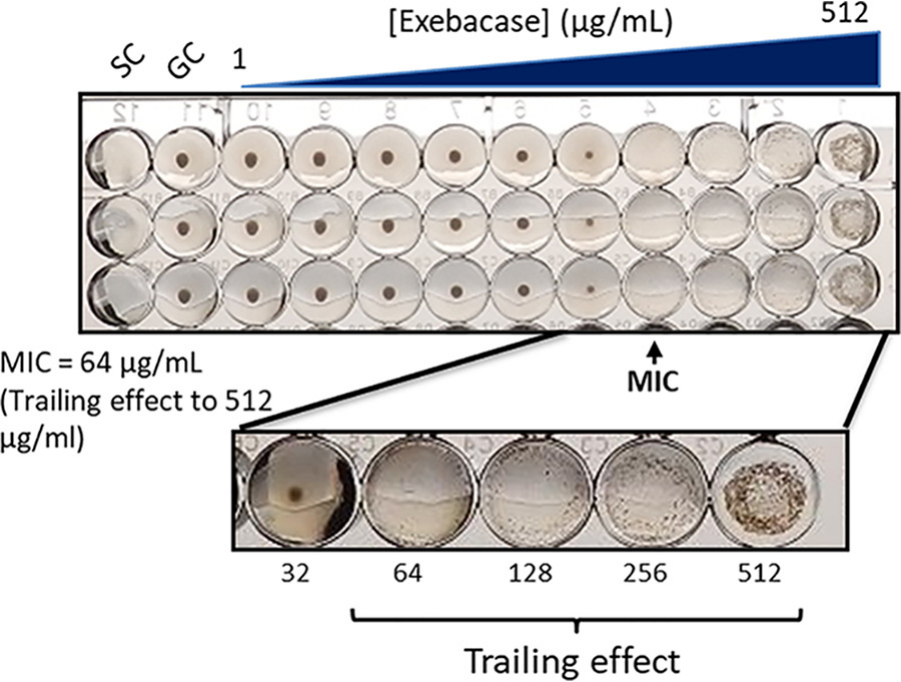
Exebacase MIC determinations against S. aureus strain NRS 123. MICs were determined (in triplicate) in CAMHB, highlighting the trailing effects at concentrations from 64 to 512 μg/ml. GC, growth control; SC, sterility control.

Exebacase MICs were determined in triplicate using CAMHB alone against a discrete heterogenous collection of 25 S. aureus (methicillin-susceptible S. aureus [MSSA] and methicillin-resistant S. aureus [MRSA]) isolates, including various clinical and laboratory-derived resistant phenotypes (see Table S1 in the supplemental material) in accordance with CLSI M07-A11 recommendations (9). Exebacase MICs generated with CAMHB alone ranged from 2 to 128 μg/ml, and MIC_50_ and MIC_90_ values were 32 and 64 μg/ml (Table 1). MIC endpoints were obscured by trailing for all of the isolates tested, including the QC strains, as illustrated by the community-acquired methicillin-resistant S. aureus strain NRS 123 (USA 400) (Fig. 1). Marked trailing was evident over a range of exebacase concentrations at and above the MIC endpoint of 64 μg/ml.

**TABLE 1:** Impact of BMD assay modifications and supplements on exebacase MICs against 25 S. aureus isolates^*a*^

Supplementation/modification	MIC (μg/ml)			Trailing effect
	50%	90%	Range	
None	32	64	2–128	Yes
0.002% polysorbate 80	64	64	8–64	Yes
50 μg/ml Ca^2+^	32	64	1–128	Yes
2% NaCl	128	128	32–128	Yes
2.5% laked horse blood	32	64	4–256	Yes
5% laked horse blood	32	64	4–256	Yes
10% laked horse blood	16	32	2–128	Yes
25% laked horse blood	8	16	2–128	Yes
Polypropylene plates	16	128	4–128	Yes
0.1% BSA (polypropylene plates)	8	32	1–32	Yes
0.1% BSA	8	32	4–32	Yes
0.1% BSA, 2% NaCl, 200 rpm	32	128	8–>256	Yes
0.1% BSA, 2% NaCl	32	64	16–128	Yes
CO_2_ atmosphere	32	64	32–128	Yes
0.5 mM DTT	128	128	8–256	Yes
50% horse serum	0.5	2	0.125–2	No
25% horse serum	0.5	2	0.125–2	No
12.5% horse serum	1	4	0.125–4	No
6.25% horse serum	4	8	1–8	No

a MICs were determined by broth microdilution in 96-well, round-bottom, polystyrene microtitration plates (unless otherwise indicated) according to the method described in CLSI document M07-A11 (9), with the indicated supplements and modifications.

Moreover, exebacase activity was diminished when frozen BMD MIC panels were used. The impact of freeze-thaw conditions was assessed by replicating conditions associated with the preparation and use of frozen BMD panels in-house or by a commercial manufacturer to support the susceptibility testing of clinical trial isolates by a central microbiology laboratory used in clinical trials (15). Using thawed exebacase BMD panels, originally prepared and frozen in CAMHB at −80°C until use, MICs ranged from 32 to >256 μg/ml and MIC_50_ and MIC_90_ values were 128 and 256 μg/ml against the same collection of 25 clinical S. aureus isolates (Table 2). The reduction in exebacase activity seen with thawed BMD panels compared to freshly prepared BMD panels was marked (approximately 2- to 16-fold).

**TABLE 2:** Impact of a freeze-thaw cycle on exebacase MICs against 25 S. aureus isolates

Medium^*a*^	Freeze-thaw	MIC (μg/ml)			Trailing
		50%	90%	Range	effect
CAMHB	No	32	64	2–128	Yes
	Yes	128	256	32–>256	Yes
CAMHB, 25% horse serum	No	0.5	2	0.125–2	No
	Yes	2	2	0.5–8	No
CAMHB, 0.5 mM DTT	No	128	128	8–256	Yes
	Yes	128	256	64–>256	Yes
CAMHB, 25% horse serum, 0.5 mM DTT	No	0.5	1	0.25–2	No
	Yes	0.5	1	0.25–2	No

a Medium was prepared using reagents from the following sources: CAMHB (BBL Mueller-Hinton broth II, cation adjusted), Becton Dickinson, catalog number 212322; horse serum (donor herd), Sigma-Aldrich, catalog number H1270; and DTT, Sigma-Aldrich, catalog number 646563.

### Standard CLSI M23-A4 supplements do not reduce trailing.

To eliminate or reduce trailing and thereby improve accurate MIC determination, MIC testing was conducted using CAMHB supplemented with either polysorbate 80, Ca^2+^, NaCl, or laked horse blood as described elsewhere (8, 16). None of the standard supplements tested diminished trailing (Table 1 and data not shown). In addition, testing was performed using polypropylene plates with and without bovine serum albumin in place of polystyrene plates (as described for cationic antimicrobial peptides to reduce adherence to plastic surfaces [17, 18]); however, their use did not reduce trailing. Supplementation of CAMHB with either bovine serum albumin alone or with NaCl (with and without agitation at 200 rpm), as described for lysostaphin MIC determination (19), also did not impact trailing, nor did the incubation of assay plates in a 5% CO_2_ atmosphere at 35°C ± 2°C or supplementation of CAMHB with either 25 mM or 44 mM NaHCO_3_, as described by Ersoy et al. (20).

### Supplementation with horse serum eliminates trailing.

We previously reported a marked (∼64-fold) reduction in exebacase MICs observed in the presence of human serum compared to MICs generated in CAMHB alone (28). Based on these findings and taken with the precedent of using horse blood in AST of Streptococcus spp. (12), the impact of horse serum on exebacase AST was examined against the collection of 25 S. aureus isolates. The use of horse serum resulted in an exebacase MIC range of 0.125 to 2 μg/ml (MIC_50_ and MIC_90_ values of 0.5 and 2 μg/ml) and eliminated the trailing when diluted in CAMHB to concentrations of at least 25% (Table 1). Horse serum concentrations at or below 12.5% resulted in MIC increases greater than 2 log_2_ dilutions compared to values obtained with higher concentrations of horse serum.

### dl-Dithiothreitol stabilizes exebacase activity in CAMHB with 25% horse serum.

The use of CAMHB supplemented with 25% horse serum remained hampered by a loss of activity when frozen exebacase BMD panels were used, compared to freshly prepared BMD panels. The thawed BMD panels yielded MIC_50_ and MIC_90_ values of 2 and 2 μg/ml (MIC range of 0.5 to 8 μg/ml) for the 25 S. aureus isolates, compared to MIC_50_ and MIC_90_ values of 0.5 and 2 μg/ml (MIC range of 0.125 to 2 μg/ml) obtained with freshly prepared BMD panels (Table 2).

**FIG 2:**
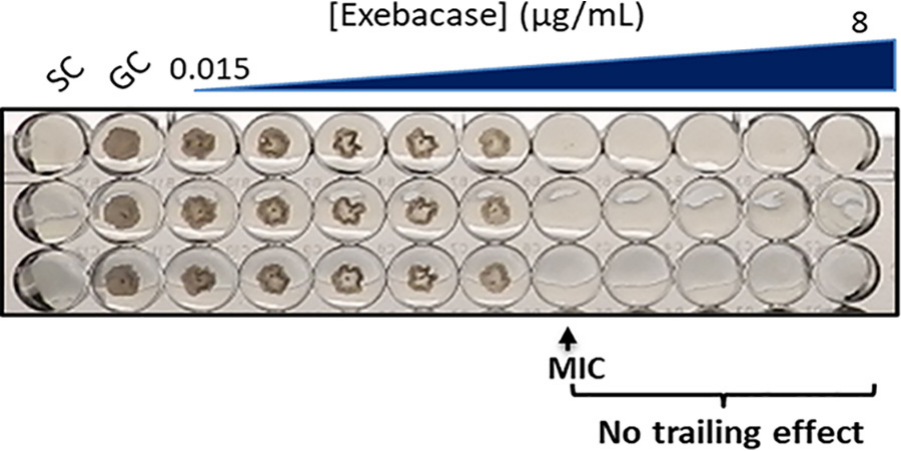
MIC determination in triplicate against S. aureus strain NRS 123 in CAMHB-HSD. A clear and unambiguous MIC is observed at 0.5 μg/ml. GC, growth control; SC, sterility control.

To stabilize exebacase activity after thawing, we considered the enzymatic nature of exebacase and the need to protect its active-site cysteine residue from air oxidation and inactivation using the reducing agent dl-dithiothreitol (DTT). Notably, DTT protects the catalytic site cysteines of thiol enzymes, like exebacase, by preventing the oxidation of sulfhydryl groups (21). When exebacase activity against S. aureus strain NRS 123 was determined in CAMHB supplemented with both 25% horse serum and a range of DTT concentrations (0.01 to 2 mM), the activity was stabilized (i.e., there was no difference in observed MICs obtained using freshly prepared CAMHB-HSD medium versus previously frozen CAMHB-HSD medium) at DTT concentrations of ≥0.5 mM (Table S2). When the collection of 25 S. aureus isolates was tested using CAMHB supplemented with 25% horse serum and 0.5 mM DTT (CAMHB-HSD) and results were compared to MIC data generated using freshly prepared BMD panels, no differences in exebacase MIC ranges or MIC_50_ and MIC_90_ values were observed (Table 2). An example of clear exebacase MIC endpoints in CAMHB-HSD against strain NRS 123 are shown in Fig. 2. Supplementation with DTT alone (i.e., without 25% horse serum), however, resulted in much higher MIC_50_ and MIC_90_ values for both freshly prepared and frozen/thawed BMD panels (128 and 128 μg/ml and 128 and 256 μg/ml, respectively), and, for this reason, supplementation with both 25% horse serum and 0.5 mM DTT is required.

### Growth rates in CAMHB-HSD and CAMHB are similar.

The growth kinetics of the CLSI reference QC strain S. aureus ATCC 29213 in CAMHB-HSD and in CAMHB alone were compared. Over a 24-h period, aerated cultures grown in CAMHB-HSD and in CAMHB alone demonstrated equivalent growth kinetics (Fig. S1). Furthermore, the doubling times in CAMHB-HSD and CAMHB were similar, at 39 and 41 min, respectively.

### Low MIC variability associated with different commercial sources of CAMHB and DTT.

The use of two different commercial sources of dehydrated CAMHB and preprepared CAMHB generated similar MIC data: MIC_50_ and MIC_90_ of 0.5 and 1 μg/ml (MIC range of 0.25 to 2 μg/ml) and MIC_50_ and MIC_90_ of 1 and 1 μg/ml (MIC range of 0.5 to 2 μg/ml), respectively (Table S3). Different commercial sources of DTT were likewise found to have little impact on MIC variability, with MIC_50_ and MIC_90_ (and range) values for both freshly prepared and frozen/thawed panels of 0.5 and 1 μg/ml (0.25 to 2 μg/ml) (Table S4).

### Low MIC variability associated with different testing conditions using CAMHB-HSD.

The impact of variations in medium pH, bacterial inoculum, and incubation duration and conditions (temperature and CO_2_) were investigated using the 25 clinical S. aureus isolates. Standard testing conditions in CAMHB-HSD relied on a medium pH value of 7.4, a bacterial inoculum of 5 × 10^5^ CFU/ml, and incubation at 35°C ± 2°C in ambient air for 18 h; these conditions resulted in MIC_50_ and MIC_90_ values of 0.5 and 1 μg/ml (MIC range of 0.25 to 2 μg/ml) (Table S5). Variations in medium pH (including pH values of 7.0 and 8.0), bacterial inoculum (5 × 10^4^, 1 × 10^5^, and 5 × 10^6^), incubation duration (24 and 48 h), and incubation conditions (ambient air at 30°C and in a 5% CO_2_ atmosphere at 35°C ± 2°C) yielded very similar MIC data with differences of <2 log_2_ dilutions. Exebacase MIC determination in CAMHB-HSD is not overtly impacted by minor methodological variations.

### Twelve-month stability of exebacase in frozen BMD panels.

Freshly prepared exebacase BMD panels were frozen promptly at −80°C and thawed at multiple intervals for up to 1 year, prior to the determination of MIC endpoints against the 25 S. aureus isolates. The exebacase MIC range and MIC_50_ and MIC_90_ generated from frozen and thawed BMD panels were stable for at least 12 months, with values of either 0.5 and 0.5 μg/ml (0.25 to 1 μg/ml) or 0.5 and 1 μg/ml (0.25 to 2 μg/ml) (Table S6). No trending was observed with the MIC data generated during the 12-month study period.

### CLSI M23-defined preliminary QC study.

A CLSI M23 preliminary MIC QC tier 1 study was conducted using CAMHB-HSD (8) at two independent testing laboratories (The Clinical Microbiology Institute, Wilsonville, OR, and the ContraFect Research Laboratory, Yonkers, NY) to establish preliminary exebacase MIC QC ranges for appropriate reference QC strains, S. aureus ATCC 29213 and E. faecalis ATCC 29212. The MIC results generated by both laboratories for S. aureus ATCC 29213 and E. faecalis ATCC 29212 using two different CAMHB lots from the same commercial manufacturer are presented in Table 3. A total of 50 MIC results were generated by each testing laboratory (25 replicate results for each ATCC strain) over 6 days using two different lots of CAMHB broth supplemented with 25% horse serum and 0.5 mM dl-dithiothreitol. For S. aureus ATCC 29213, the overall distribution of MIC results was 2 log_2_ dilution steps (0.5 to 1 μg/ml), 98.0% of results at the mode (0.5 μg/ml) and 100% of results within ±1 doubling dilution of the mode. For E. faecalis ATCC 29212, the overall distribution of MIC results was 2 log_2_ dilution steps (16 to 32 μg/ml), 86.0% of results at the mode (32 μg/ml), and 100% within ±1 doubling dilution of the mode. Preliminary QC ranges of 0.25 to 1 μg/ml for S. aureus ATCC 29213 and 16 to 64 μg/ml for E. faecalis ATCC 29212 were identified.

**TABLE 3:** Medium lot and inter- and intralaboratory comparisons of exebacase MICs obtained against QC strains S. aureus ATCC 29213 and E. faecalis ATCC 29212

Strain and MIC (μg/ml)	No. of occurrences for:				
	CAMHB lot		Each laboratory		
	Lot 1	Lot 2	Lab 1	Lab 2	Total, mean, or mode
ATCC 29213^*a*^					
0.5	24	25	24	25	49
1	1		1		1

Total	25	25	25	25	50
Geometric mean	0.51	0.50	0.51	0.50	0.51
Mode	0.50	0.50	0.50	0.50	0.50
Log_2_ range	2	1	2	1	2

ATCC 29212^*b*^					
16	7		7		7
32	18	25	18	25	43

Total	25	25	25	25	50
Geometric mean	26.35	32.0	26.35	32.0	29.04
Mode	32	32	32	32	32
Log_2_ range	2	1	2	1	2

a The MICs shown represent the preliminary QC range of 0.25 to 1 μg/ml for ATCC 29213. No occurrences were observed for MICs of 0.25 and 2 μg/ml.

b The MICs shown represent preliminary QC range of 16 to 64 μg/ml for ATCC 29212. No occurrences were observed for MICs of 8 and 64 μg/ml.

### Exebacase *in vitro* activity against 149 clinical S. aureus isolates.

The *in vitro* activity of exebacase was determined against a previously described collection of clinical S. aureus (74 MSSA and 75 MRSA) isolates using frozen BMD CAMHB-HSD panels. The exebacase MIC distribution ranged from 0.12 to 1 μg/ml, the modal MIC of 0.5 μg/ml represented 83% of the isolates tested, and MIC_50_ and MIC_90_ values were 0.5 and 0.5 μg/ml and 0.5 and 1 μg/ml for MSSA and MRSA isolates, respectively (Table 4) and are consistent with data we reported previously for these isolates tested in undiluted human serum (28).

**TABLE 4:** Exebacase MICs determined in CAMHB-HSD using frozen-form panels

Organism	*n*	No. with exebacase MIC (μg/ml)^*a*^				MIC (μg/ml)		
		0.12	0.25	0.5	1	50%	90%	Range
S. aureus	149	1	5	124	19	0.5	1	0.125–1
MSSA	74	1	4	62	7	0.5	0.5	0.125–1
MRSA	75		1	62	12	0.5	1	0.5–1

a No isolates had MICs of 0.06 or 2 μg/ml.

### Investigation of MIC variability using different commercial sources of horse serum.

At the request of the CLSI Subcommittee on AST, a CLSI-style tier 1 QC study was conducted to evaluate various commercial sources of horse serum on the test performance of the CAMHB-HSD medium in light of significant interlaboratory variability associated with antibiotic susceptibility testing of pneumococci (22, 23). Multiple horse serum samples were tested (see Table S7). Five replicates of each QC strain were tested each day, for 5 separate days, i.e., a total of 25 replicates of each serum sample, generating a total of 350 exebacase MIC results for each QC organism. For S. aureus ATCC 29213, 98.6% of MICs were within ±1 doubling dilution of the mode (0.5 μg/ml), and for E. faecalis ATCC 29212, 100% were within ±1 doubling dilution of the mode (32 μg/ml) (Table 5). Importantly, CAMHB-HSD medium prepared with each of the 14 horse serum samples produced MIC ranges within the exebacase QC ranges established in a multilaboratory QC tier 1 and subsequent tier 2 study (12).

**TABLE 5:** Exebacase QC MIC distributions of S. aureus (ATCC 29213) and E. faecalis (ATCC 29212) in 14 lots of horse serum determined at Clinical Microbiology Institute (Wilsonville, OR)

Strain and MIC (μg/ml)	No. of occurrences with horse serum lot^*a*^														
	1	2	3	4	5	6	7	8	9	10	11	12	13	14	Total
ATCC 29213															
0.25												3	11	17	31
0.5	24	13	24	21	8		16	24	23	25	25	21	13	8	245
1	1	12	1	4	17	20	9	1	2			1	1		69
2						5									5

Total (*n*)	25	25	25	25	25	25	25	25	25	25	25	25	25	25	350
Geometric mean	0.52	0.74	0.52	0.58	0.84	1.2	0.68	0.52	0.54	0.5	0.5	0.49	0.41	0.33	0.59
Mode	0.5	0.5	0.5	0.5	1	1	0.5	0.5	0.5s	0.5	0.5	0.5	0.25	0.25	0.5
Range	2	2	2	2	2	2	2	2	2	1	1	3	3	2	4

ATCC 29212															
8															0
16		3	15		1	22	1	24	10	22	23		23	18	162
32	25	22	10	25	23	3	23	1	15	3	2	25	2	7	186
64					1		1								2

Total (*n*)	25	25	25	25	25	25	25	25	25	25	25	25	25	25	350
Geometric mean	32	30.1	22.4	32	32.6	17.9	32.6	16.6	25.6	17.9	17.2	32	17.3	20.4	24.8
Mode	32	32	16	32	32	16	32	32	16	16	16	16	16	16	16
Range	1	2	2	1	1	2	3	2	2	2	2	1	2	2	3

a The source of each of the 14 horse serum samples is indicated in Table S7.

## DISCUSSION

Lysins are a novel class of antibacterial agents, distinct from conventional antibiotics with respect to chemical/physical characteristics and an enzymatic mechanism of action based on rapid cell wall hydrolysis and osmotic lysis. Consideration of these differences gave rise to the unique modifications tested in our studies to establish a new reference BMD method for accurate and reproducible MIC determination. The use of horse serum at a concentration of 25% specifically abrogated the distinctive trailing effect observed when CAMHB alone was used and yielded MIC data equivalent to data generated using human serum alone. Supplementation with horse serum enabled a better understanding of antistaphylococcal activity in a biologically relevant environment, compared to MIC data generated using CAMHB alone.

While the inclusion of 25% horse serum in CAMHB-HSD mitigated the observed trailing MIC endpoints and thereby facilitated the reading of exebacase MIC endpoints, the inclusion of 0.5 mM DTT served to stabilize exebacase and thereby facilitate the long-term storage (up to 12 months) of frozen BMD panels. The mechanism of stabilization is understood in light of the enzymatic nature of exebacase, which is a cysteine/histidine-dependent amidohydrolase/peptidase with an active-site cysteine residue that is subject to air oxidation (3, 24). A strong reducing agent such as DTT likely prevents oxidation of sulfhydryl groups in cysteine that may occur during the handling of frozen exebacase BMD panels. DTT is a critical supplement to facilitate large-scale MIC panel preparation by a central laboratory for testing clinical isolates from antibacterial surveillance programs and clinical trials.

As the first AST medium to be described for MIC determination of a lysin enzyme, CAMHB-HSD allows accurate and reproducible MIC determination, and concomitant QC testing allows evaluation of test performance. The medium provided consistent, reproducible MIC results across a range of clinical S. aureus isolates tested, including various resistant phenotypes, and across a variety of testing conditions. Variations in the commercial source of individual medium components, including CAMHB, DTT, and horse serum, had no impact on MICs determined using either freshly prepared or frozen BMD panels. There was, likewise, no impact on MICs associated with variations tested in medium pH, culture inoculum, incubation temperature and atmosphere, or incubation length. Importantly, MICs obtained using BMD panels stored for up to 12 months at −80°C exhibited minor MIC variation, of ≤1 log_2_ dilution.

The BMD method described herein and expected exebacase QC ranges identified from a subsequent multilaboratory CLSI M23-defined MIC tier 2 study (0.25 to 2 μg/ml for S. aureus ATCC 29213 and 8 to 64 μg/ml for E. faecalis ATCC 29212) are now published in CLSI M100 (12). The BMD method and concomitant QC testing were used to evaluate exebacase susceptibility against clinical S. aureus isolates in a recent surveillance study (25) and in the completed phase 2 clinical trial (NCT03163446) (26). The test method will furthermore be used to support antibacterial surveillance studies and the ongoing superiority designed phase 3 (NCT04160468) DISRUPT study to evaluate the efficacy and safety of exebacase in addition to antistaphylococcal antibiotics compared with antibiotics alone for the treatment of S. aureus bloodstream infections, including right-sided infective endocarditis (27).

## MATERIALS AND METHODS

### Bacterial strains.

The discrete collection of 25 S. aureus strains tested in CAMHB and subsequently used throughout the study to identify a suitable medium for testing exebacase susceptibility and assess the influence of various parameters known to impact MIC results is presented in Table S1. The collection of 149 clinical S. aureus isolates (74 MSSA and 75 MRSA) used to validate the test performance of the exebacase AST medium was obtained from JMI Laboratories (North Liberty, IA) and was described previously (5, 28). Staphylococcus aureus ATCC 29213 and Enterococcus faecalis ATCC 29212 were selected as reference QC strains for M23-style tier 1 studies (8).

### Materials and chemicals.

Exebacase was obtained from ContraFect Corporation (lot number NBA0467-08). Growth media (and sources) are as follows: BBL Mueller-Hinton broth II, cation adjusted (Becton, Dickinson and company [BD]; catalog number 296164, lot number 6054984); BBL Mueller-Hinton II broth, cation adjusted (BD; catalog number 212322, lot numbers 2089488 and 5257869); BBL Mueller-Hinton II broth, cation adjusted (BD; catalog number 297963, lot numbers 6014547 and 4044343); BBL Mueller-Hinton broth II (Sigma-Aldrich; catalog number 90922, lot number BCBR3303V); and BBL Mueller-Hinton II broth, cation adjusted (Teknova; catalog number M5860, lot number M586012B1601). Horse serum was obtained from Sigma-Aldrich (catalog number H1270, lot number 15G382), BioreclamationIVT (catalog number HSESRM, lot number HSE1225), Gibco (catalog number 16050-122, lot number 1671315), and Corning (catalog number 35-030-CV, lot number 35030105). Laked horse blood was obtained from Hema Resource and Supply, Inc. (catalog number 15-14-0050-28). Additional sources of horse serum are described in Table S7. Bovine serum albumin (catalog number A2153, lot number SLBL5462V), dl-dithiothreitol solution (catalog number 646563, lot numbers MKBW5575V and MKBX878V), dl-dithiothreitol (catalog number 43815-25G, lot number BCBBD7009V), and Tween 80 (catalog number P4780, lot number MKBW2896V) were obtained from Sigma-Aldrich. dl-Dithiothreitol was also obtained from G-Biosciences (catalog number RC-046, lot number 151106). Sodium chloride (catalog number BP358-10, lot number 150661) and calcium chloride (catalog number C77-212, lot number 110651) were obtained from Fisher Scientific.

### MIC determination by broth microdilution.

Assays were performed according to CLSI M07-A11 guidelines (9) in triplicate, with the exception of the indicated procedural variations and use of nonstandard AST media. The experimental parameters were varied one at a time during testing, and results were compared with those obtained using the standard reference BMD method (CAMHB). Falcon 96-well polystyrene plates (Corning; catalog number 351177) were used in all assays, except for indicated analyses using Falcon 96-well polypropylene plates (Corning; catalog number 351190). Assay plates were incubated for 18 h at 35 ± 2°C in ambient air, and MICs were determined visually unless otherwise indicated. To investigate the impact of freeze-thawing on exebacase activity in dilution panels (in indicated media), plates were transferred to a −80°C freezer within 2 h of preparation; after 24 h (unless otherwise indicated), the plates were removed and thawed for 1 h in ambient air prior to the addition of inocula (final cell density = 5 × 10^5^ CFU/ml) and overnight incubation at 35°C ± 2°C to determine MICs.

### Preparation of CAMHB-HSD.

The CAMHB was prepared and stored according to each of the manufacturer’s recommendations. Horse serum was stored frozen at −20°C and thawed in a 24°C water bath for 30 min prior to use. After thawing, the horse serum was aseptically added to CAMHB to a final concentration of 25% and swirled to mix. The dl-dithiothreitol was then added aseptically to a final concentration of 0.5 mM and swirled to mix. Broth pH was typically between 7.2 and 7.4 after the supplementation. The CAMHB-HSD medium was used within 2 h of preparation.

### Growth kinetics in CAMHB-HSD.

The growth of S. aureus strain ATCC 29213 was examined in both the reference BMD medium (CAMHB) and CAMHB-HSD. An overnight culture was diluted by a factor of 1:100 into each medium and incubated at 35°C ± 2°C with agitation at 150 rpm for 24 h. Culture samples were collected every hour for 2 h and then every 30 min for the next 6 h (with a final collection at 24 h). For each time point, optical density (OD) at 600 nm was recorded. The optical densities were plotted on a log_10_ scale. Each condition was examined in triplicate, and mean values with standard deviation are shown. Culture doubling times were calculated exactly as described elsewhere (29).

### CLSI M23-A5 tier 1 study.

Testing was performed at the Clinical Microbiology Institute (Wilsonville, OR) and at ContraFect (Yonkers, NY). Each lab used a unique lot of CAMHB II from Becton, Dickinson and Company (Clinical Microbiology Institute, lot number 4044343; ContraFect, lot number 20894988). The broths were autoclaved, cooled, and then supplemented with 25% horse serum (Sigma-Aldrich, lot number 15G382) plus 0.5 mM DTT (dl-dithiothreitol solution) (Sigma-Aldrich; lot number MKBW8225V) by aseptic addition. Broth pH was between 7.2 and 7.4 after additions at both laboratories. Working solution and test dilutions of exebacase were prepared in CAMHB-HSD. Vancomycin (Clinical Microbiology Institute, Sigma-Aldrich lot number SLBM3283V; ContraFect, Sigma-Aldrich lot number 120M14595V) and oxacillin (Clinical Microbiology Institute only; Sigma-Aldrich lot number 035M4889V) were tested in CAMHB or CAMHB plus 2% saline. Twenty-five replicates of two QC strains, S. aureus ATCC 29213 and E. faecalis ATCC 29212, were tested over 6 separate days. All inocula were prepared in 0.85% saline to match a McFarland 0.5 turbidity standard using colonies from a plate that has been incubated for 18 to 24 h. Each replicate was tested using a unique 0.5 McFarland preparation. Each inoculum preparation was further diluted with saline to obtain a final inoculum of approximately 5 × 10^5^ CFU/ml in the test panel. Colony counts were performed daily.

### Tier 1 CLSI QC study to investigate the test performance using horse serum from different commercial sources.

An M23-A5 tier 1 QC study was performed at the Clinical Microbiology Institute (Wilsonville, OR) using CAMHB-HSD prepared with each of 14 different sources (Table S7) of non-heat-inactivated horse serum. Single lots of both CAMHB (Becton, Dickinson and Company; lot number 5257869) and DTT (Sigma-Aldrich; lot number MKBX878V) were used. Serial 2-fold dilutions of exebacase were prepared in each of the 14 lots of CAMHB-HSD. All dilutions were dispensed into 96-well polystyrene plates and frozen at −80°C until the day of use. Vancomycin powder purchased from Sigma-Aldrich (lot number SLBM3283V) was tested using CAMHB alone. For each lot of CAMHB-HSD, 5 to 8 replicates of each of the two QC strains (S. aureus ATCC 29213 and E. faecalis ATCC 29213) were tested each day for 5 separate days for a total of 25 replicates. Separate McFarland inocula were used for each test replicate each day. Inocula were prepared in 0.85% saline and adjusted to match a McFarland 0.5 turbidity standard using colonies from blood agar plates that had been incubated at 35°C ± 2°C for 18 to 24 h. Each replicate was tested using a unique inoculum preparation. Each inoculum preparation was further diluted to obtain a final inoculum of approximate 5 × 10^5^ CFU/ml in the MIC test panel. Colony counts were performed on 1 replicate of each strain per day.
