# Supplementary material for: Development of a Broth Microdilution Method for Exebacase Susceptibility Testing
Source: Antimicrob Agents Chemother. 2021 Jun 17;65(7):e02587-20. doi: 10.1128/AAC.02587-20 (PMC8218677; doi:10.1128/AAC.02587-20)
Supplement: Supplemental file 1 — Supplemental tables and figure. Download AAC.02587-20-s0001.pdf, PDF file, 0.2 MB [file aac.02587-20-s0001.pdf]

Supplemental Material

TABLE S1 Strains used in this study

Designation	Type	Description	Source and/or Reference
ATCC 49521	MSSA	Capsule serotype V strain, bacteremia isolate	ATCC (1)
ATCC 25923	MSSA	Quality control strain	ATCC (2)
JMI 2559	MSSA	Isolated from respiratory tract infection	JMI Laboratories
JMI 3126	MSSA	Isolated from respiratory tract infection	JMI Laboratories
NRS 143	MSSA	*agr* III	BEI Resources (3)
ATCC 43300	MRSA	Reference strain, SCC*mec* type II, PVL-negative	ATCC (2)
NRS 123	MRSA	Strain MW2, SCC*mec* type IV, PVL-positive, PFGE type USA400	BEI Resources (4)
CAIRD 426	MRSA	PFGE type USA100, Hospital-acquired MRSA	David Nicholau, Center for Anti-Infective Research and Development, Hartford Hospital (5)
NRS 100	MRSA	Strain COL, SCC*mec* type I, Panton-Valentine leucocidin (PVL) toxin-negative	BEI Resources, catalog number NR-45906 (6)
JMI 227	MRSA	Clinical blood isolate, SCC*mec* type IV, PVL-negative	JMI Laboratories
CFS 954	DRSA	Daptomycin-resistant derivative (MIC=4 µg/mL) of ATCC 43300 obtained in serial passage	(7)
CFS 1239	DRSA	Daptomycin-resistant derivative (MIC=4 µg/mL) of ATCC 25923 obtained in serial passage	(7)
CFS 1238	DRSA	Daptomycin-resistant derivative (MIC=8 µg/mL) of ATCC 25923 obtained in serial passage	(7)
CFS 1237	DRSA	Daptomycin-resistant derivative (MIC=8 µg/mL) of *S. aureus* strain MW2 (NRS 123) obtained in serial passage	(7)
CFS 1233	DRSA	Daptomycin-resistant derivative (MIC=16 µg/mL) of *S. aureus*	(7)

		strain MW2 (NRS 123) obtained in serial passage	
NRS 127	LRSA	Linezolid-resistant (MIC=8 µg/mL), sputum isolate, SCC*mec* II, MLST sequence type (ST) 5, eGenomic spa type 2	BEI Resources catalog number NR-45930 (8)
NRS 271	LRSA	Linezolid-resistant (MIC=32 µg/mL), wound drain site isolate, SCC*mec* IV, MLST sequence type (ST) 22; eGenomic spa type 382	BEI Resources catalog number NR-46062 (8)
NRS 119	LRSA	Linezolid-resistant (MIC=64 µg/mL), SCC*mec* IV, MLST sequence type (ST) 507, eGenomic spa type 7	BEI Resources catalog number NR-45924 (8)
NRS 121	LRSA	Linezolid-resistant (MIC=64 µg/mL), SCC*mec* IV; MLST sequence type (ST) 507; eGenomic spa type 7	BEI Resources catalog number NR-45926 (8)
NRS 120	LRSA	Linezolid-resistant (MIC=64) µg/mL, closely related to NRS 119	BEI Resources (9)
VRS1	VRSA	Vancomycin-resistant (MIC=>32 µg/mL), infected catheter source, PFGE type USA100; MLST (ST) 5	BEI Resources catalog number NR-46410 (10)
VRS2	VRSA	Vancomycin-resistant (MIC=64 µg/mL), plantar ulcer isolate, PFGE type USA100; MLST (ST) 5	BEI Resources catalog number NR-46411 (11)
VRS3a	VRSA	Vancomycin-resistant (MIC=>32 µg/mL), nephrostomy tube exit site isolate, PFGE type USA800; MLST (ST) 5	BEI Resources catalog number NR-46412 (12)
VRS3b	VRSA	Vancomycin-resistant (MIC=>32 µg/mL), nephrostomy tube exit site isolate, PFGE type USA800; MLST (ST) 5	BEI Resources catalog number NR-46413 (12)
VRS4	VRSA	Vancomycin-resistant (MIC=>32 µg/mL), wound isolate, PFGE type USA100; MLST (ST) 5	BEI Resources catalog number NR-46414 (13)

Abbreviations = MSSA, methicillin-sensitive *S. aureus*; MRSA, methicillin-resistant *S. aureus*; DRSA, daptomycin-resistant *S. aureus*; LRSA, linezolid-resistant *S. aureus*; VRSA, vancomycin-resistant *S. aureus*; PFGE, Pulsed Field Gel Electrophoresis; PVL, Panton–Valentine Leucocidin; ATCC, American Type Culture Collection; MLST, Multilocus Sequence Type; and SCC, *Staphylococcus* Cassette Chromosome.

TABLE S2 Impact of DTT on exebacase MICs (µg/mL) against *S. aureus* strain NRS 123 in CAMHB supplemented with 25% horse serum (with and without freeze/thaw cycles)[a]

DTT concentration	MIC (no freeze/thaw)	MIC (yes freeze/thaw)	Trailing effect[b]
No DTT	1	4	No
0.01 mM	1	2	No
0.05 mM	1	2	No
0.1 mM	1	2	No
0.125 mM	1	2	No
0.25 mM	0.5/1	0.5/1	No
0.5 mM	0.5	0.5	No
1 mM	0.5	0.5	No
2 mM	0.5	0.5	No

[a]Media was prepared using reagents from the following sources: CAMHB (BBL Mueller Hinton broth II, cation-adjusted), Becton Dickenson and Co. catalog number 212322; horse serum (donor herd), Sigma-Aldrich catalog number H1270; and DTT, Sigma-Aldrich catalog number 646563.

[b]Refers to the analysis after a freeze/thaw cycle.

TABLE S3 Effect of varying CAMHB sources on exebacase MICs determined against 25 *S. aureus* strains in CAMHB-HSD[a]

CAMHB source (catalog number)	Freeze/thaw	MIC (µg/mL)		
		MIC_{50}	MIC_{90}	Range
BBL™ BD (212322)	No	0.5	1	0.25 - 2
	Yes	0.5	1	0.25 - 2
Sigma-Aldrich (90922)	No	1	2	0.5 – 2
	Yes	1	2	0.5 - 2
Teknova (M5860)	No	0.5	1	0.25 - 1
	Yes	0.5	1	0.25 - 1
BD (297963)	No	0.5	1	0.25 - 1
	Yes	0.5	1	0.25 - 1

[a]Additional media reagents were obtained from the following sources: horse serum (donor herd), Sigma-Aldrich catalog number H1270; and DTT, Sigma-Aldrich catalog number 646563.

TABLE S4 Effect of varying DTT sources on exebacase MICs determined against 25 *S. aureus* strains in CAMHB-HSD[a]

DTT source (catalog number)	Freeze/thaw	MIC (µg/mL)		
		MIC_{50}	MIC_{90}	Range
Sigma-Aldrich (646563)	No	0.5	1	0.25 - 2
	Yes	0.5	1	0.25 - 2
Sigma-Aldrich (43815-25G)	No	0.5	1	0.25 - 2
	Yes	0.5	1	0.25 - 2
G-Biosciences (RC-046)	No	0.5	1	0.25 - 2
	Yes	0.5	1	0.25 - 2

[a]Additional media reagents were obtained from the following sources: CAMHB (BBL Mueller Hinton broth II, cation-adjusted), Becton Dickenson and Co. catalog number 212322; and horse serum (donor herd), Sigma-Aldrich catalog number H1270.

TABLE S5 Effect of varying pH, bacterial inoculum, and incubation conditions on exebacase MICs determined against 25 *S. aureus* strains in CAMHB-HSD[a]

**Condition**	**Modification**	**MIC_{50}**	**MIC_{90}**	**Range**
Final pH	Unadjusted (pH 7.4)	0.5	1	0.25-2
	Adjusted to pH 7.0	0.5	1	0.25-2
	Adjusted to pH 8.0	1	1	0.25-2
Bacterial inoculum	$5x10^6$ CFU/mL	0.5	1	0.25-1
	$5x10^5$ CFU/mL	0.5	1	0.25-2
	$1x10^5$ CFU/mL	0.5	1	0.25-2
	$5x10^4$ CFU/mL	0.5	1	0.25-1
Incubation conditions	Ambient air (37°C)	0.5	1	0.25-2
	Ambient air (30°C)	0.5	1	0.25-1
	5% CO2 (37°C)	0.5	1	0.25-2
Incubation length	18 hours	0.5	1	0.25-2
	24 hours	0.5	1	0.25-1
	48 hours	0.5	1	0.25-2

[a]Media was prepared using reagents from the following sources: CAMHB (BBL Mueller Hinton broth II, cation-adjusted), Becton Dickenson and Co. catalog number 212322; horse serum (donor herd), Sigma-Aldrich catalog number H1270; and DTT, Sigma-Aldrich catalog number 646563.

Table S6 Impact of long-term storage of exebacase dilution panels (in CAMHB-HSD) at -80°C on MICs determined against a set of 25 *S. aureus* strains[a]

Storage time (days)[a]	MIC_{50}	MIC_{90}	Range
1	0.5	0.5	0.25-1
3	0.5	1	0.25-2
7	0.5	0.5	0.25-1
14	0.5	1	0.25-2
21	0.5	1	0.25-2
28	0.5	1	0.25-2
60	0.5	0.5	0.25-2
90	0.5	1	0.25-2
120	0.5	1	0.25-2
180	0.5	0.5	0.25-1
365	0.5	0.5	0.25-2

[a]Frozen panels were prepared using reagents from the following sources: CAMHB (BBL Mueller Hinton broth II, cation-adjusted), Becton Dickenson and Co. catalog number 212322; horse serum (donor herd), Sigma-Aldrich catalog number H1270; and DTT, Sigma-Aldrich catalog number 646563.

Table S7 Horse serum samples used in M23-A5 Tier 1 study[a]

Sample number	Source	Catalog number	Lot number	Date of draw	Collection	Sex	Breed
1	Sigma-Aldrich	H1270-500ML	15G382	Summer 2015	Donor Herd, USA	Mixed	Mixed
2	Corning	35-030-CV	35030105	Fall 2014	Donor Equines, USA	Male (Fixed)	Belgians and Percherons
3	Sigma-Aldrich	12449C	14A277	Winter 14	Donor Herd, USA	Mixed	Mixed
4	RMBIO	DES-BBT-5XM	20160601ES	Spring 2016	Donor Herd, USA	Mixed	n.a.
5	RMBIO	DES-BBT-5XM	20150520ES	Spring 2015	Donor Herd, USA	Mixed	n.a.
6	RMBIO	DES-BBT-5XM	20160113ES	Winter 2015	Donor Herd, USA	Mixed	n.a.
7	ATCC	30-2040	62724716	Winter 2014/5	Donor Herd, USA	Male	Mixed
8	CBI	B63538	1-167-A	Winter 2016	Donor Herd, USA	Male (fixed)	Belgians, Percherons, Clydesdales
9	CBI	B63539	2-167-B	Spring 2016	Donor Herd, USA	Male (fixed)	Belgians, Percherons, Clydesdales
10	CBI	B63540	3-167-C	Summer 2016	Donor Herd, USA	Male (fixed)	Belgians, Percherons, Clydesdales
11	CBI	B63541	4-167-D	Fall 2015	Donor Herd, USA	Male (fixed)	Belgians, Percherons, Clydesdales
12	CBI	B63542	5-167-E	Summer 2016	1 individual, USA	Male (fixed)	Belgians, Percherons, Clydesdales
13	Lampire	n.a.	16H29000	Spring 2016	1 individual, USA	Female	Thoroughbred
14	Lampire	n.a.	16H29001	Fall 2015	1 individual, USA	Female	Thoroughbred

[a]Abbreviations = RMBIO, Rocky Mountain Biologicals, Inc.; ATCC, American Type Culture Collection; CBI, Central Biomedia Inc.; and n.a., not available.

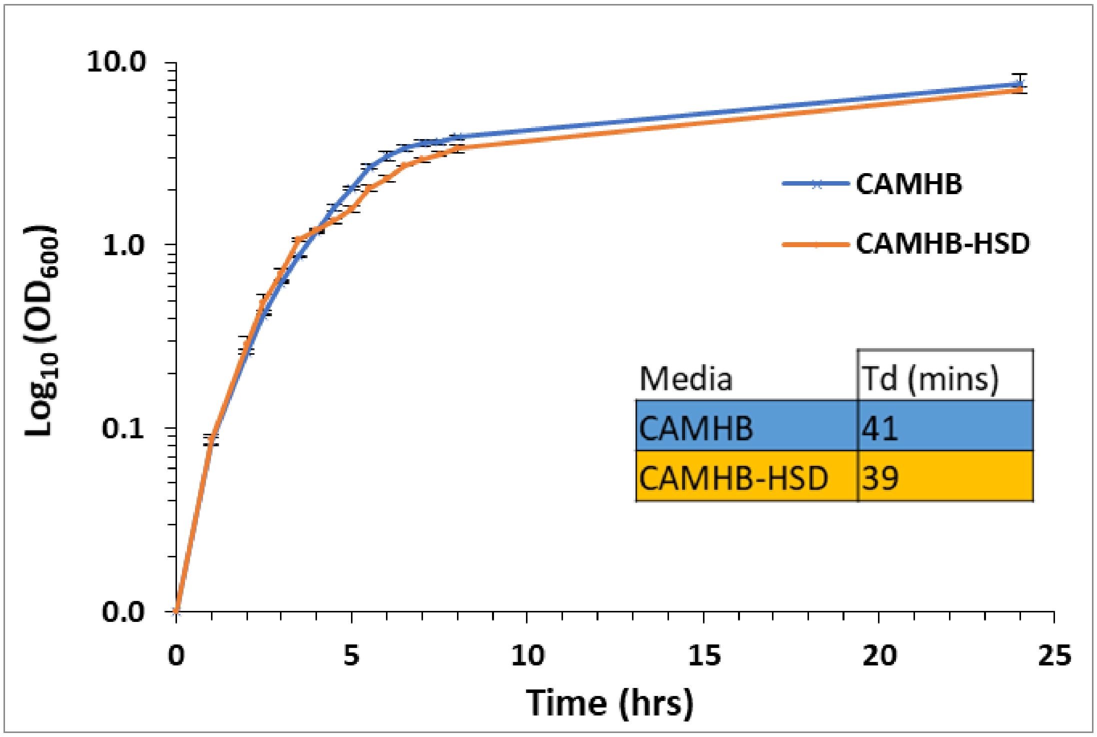

FIG S1 Growth kinetics of *S. aureus* strain ATCC 29213 cultured in CAMHB and CAMHB-HSD media. The analysis in each media type was performed in triplicate and each point is represented as the mean ± standard deviation. The doubling time (Td) for each culture in exponential phase is indicated.

REFERENCES

1. Fattom AI, Sarwar J, Ortiz A, Naso R. 1996. A *Staphylococcus aureus* capsular polysaccharide (CP) vaccine and CP-specific antibodies protect mice against bacterial challenge. Infect Immun 64:1659-65.
2. CLSI. 2019. Performance Standards for Antimicrobial Susceptibility Testing, Document M100, 29th Ed. Clinical and Laboratory Standards Institute, Wayne, PA.
3. Welsh KJ, Abbott AN, Lewis EM, Gardiner JM, Kruzel MC, Lewis CT, Mohr JF, Wanger A, Armitige LY. 2010. Clinical characteristics, outcomes, and microbiologic features associated with methicillin-resistant *Staphylococcus aureus* bacteremia in pediatric patients treated with vancomycin. J Clin Microbiol 48:894-9.
4. Baba T, Bae T, Schneewind O, Takeuchi F, Hiramatsu K. 2008. Genome sequence of *Staphylococcus aureus* strain Newman and comparative analysis of staphylococcal genomes: polymorphism and evolution of two major pathogenicity islands. J Bacteriol 190:300-10.
5. Keel RA, Tessier PR, Crandon JL, Nicolau DP. 2012. Comparative efficacies of human simulated exposures of tedizolid and linezolid against *Staphylococcus aureus* in the murine thigh infection model. Antimicrob Agents Chemother 56:4403-7.
6. Gill SR, Fouts DE, Archer GL, Mongodin EF, Deboy RT, Ravel J, Paulsen IT, Kolonay JF, Brinkac L, Beanan M, Dodson RJ, Daugherty SC, Madupu R, Angiuoli SV, Durkin AS, Haft DH, Vamathevan J, Khouri H, Utterback T, Lee C, Dimitrov G, Jiang L, Qin H, Weidman J, Tran K, Kang K, Hance IR, Nelson KE, Fraser CM. 2005. Insights on evolution of virulence and resistance from the complete genome analysis of an early methicillin-resistant *Staphylococcus aureus* strain and a biofilm-producing methicillin-resistant *Staphylococcus epidermidis* strain. J Bacteriol 187:2426-38.
7. Schuch R, Lee HM, Schneider BC, Sauve KL, Law C, Khan BK, Rotolo JA, Horiuchi Y, Couto DE, Raz A, Fischetti VA, Huang DB, Nowinski RC, Wittekind M. 2014. Combination therapy with lysin CF-301 and antibiotic is superior to antibiotic alone for treating methicillin-resistant *Staphylococcus aureus*-induced murine bacteremia. J Infect Dis 209:1469-78.
8. Silva-Del Toro SL, Greenwood-Quaintance KE, Patel R. 2016. In vitro activity of tedizolid against linezolid-resistant staphylococci and enterococci. Diagn Microbiol Infect Dis 85:102-4.
9. Tsiodras S, Gold HS, Sakoulas G, Eliopoulos GM, Wennersten C, Venkataraman L, Moellering RC, Ferraro MJ. 2001. Linezolid resistance in a clinical isolate of *Staphylococcus aureus*. Lancet 358:207-8.
10. Centers for Disease C, Prevention. 2002. *Staphylococcus aureus* resistant to vancomycin--United States, 2002. MMWR Morb Mortal Wkly Rep 51:565-7.
11. Centers for Disease C, Prevention. 2002. Vancomycin-resistant *Staphylococcus aureus*--Pennsylvania, 2002. MMWR Morb Mortal Wkly Rep 51:902.
12. Centers for Disease C, Prevention. 2004. Vancomycin-resistant *Staphylococcus aureus*--New York, 2004. MMWR Morb Mortal Wkly Rep 53:322-3.
13. Sievert DM, Rudrik JT, Patel JB, McDonald LC, Wilkins MJ, Hageman JC. 2008. Vancomycin-resistant *Staphylococcus aureus* in the United States, 2002-2006. Clin Infect Dis 46:668-74.
